# Fluorescence-Based Comparative Binding Studies of the Supramolecular Host Properties of PAMAM Dendrimers Using Anilinonaphthalene Sulfonates: Unusual Host-Dependent Fluorescence Titration Behavior

**DOI:** 10.3390/s100404053

**Published:** 2010-04-21

**Authors:** Natasa Stojanovic, Laurel D. Murphy, Brian D. Wagner

**Affiliations:** Department of Chemistry, University of Prince Edward Island, Charlottetown, PE C1A 4P3, Canada; E-Mails: natasastojanovic@yahoo.ca (N.S.); laurelmurphy@gmail.com (L.M.)

**Keywords:** PAMAM dendrimers, fluorescence enhancement, fluorescence titration curves, host-guest inclusion, anilinonaphthalene sulfonate probes

## Abstract

This work describes the fluorescence enhancement of the anilinonaphthalene sulfonate probes 1,8-ANS, 2,6-ANS, and 2,6-TNS via complexation with PAMAM dendrimer hosts of Generation 4, 5 and 6. The use of this set of three very closely related probes allows for comparative binding studies, with specific pairs of probes differing only in shape (1,8-ANS and 2,6-ANS), or in the presence of a methyl substituent (2,6-TNS *vs.* 2,6-ANS). The fluorescence of all three probes was significantly enhanced upon binding with PAMAM dendrimers, however in all cases except one, a very unusual spike was consistently observed in the host fluorescence titration plots (fluorescence enhancement *vs*. host concentration) at low dendrimer concentration. This unprecedented fluorescence titration curve shape makes fitting the data to a simple model such as 1:1 or 2:1 host: guest complexation very difficult; thus only qualitative comparisons of the relative binding of the three guests could be made based on host titrations. In the case of G4 and G5 dendrimers, the order of binding strength was qualitatively determined to be 1,8-ANS < 2,6-ANS indicating that the more streamlined 2,6-substituted probes are a better match for the dendrimer cavity shape than the bulkier 1,8-substituted probe. This order of binding strength was also indicated by double fluorometric titration experiments, involving both host and guest titrations. Further double fluorometric titration experiments on 2,6-ANS in G4 dendrimer revealed a host concentration-dependent change in the nature of the host: guest complexation, with multiple guests complexed per host molecule at very low host concentrations, but less than one guest per host at higher concentrations.

## 1. Introduction

**Figure 1.**
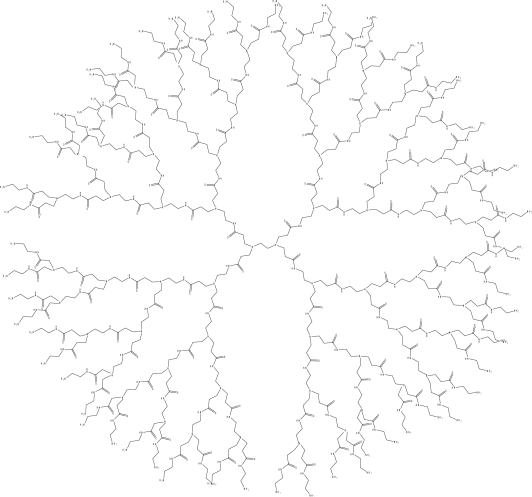
Structure of PAMAM dendrimer, Generation 4.

Dendrimers are an interesting class of macromolecules that have received significant attention in recent years due to their highly branched structure and the uniformity of their size and shape [1–4]. They possess high concentrations of functional groups and internal cavities, and their unique structure and size range have a great impact on their physical and chemical properties, as well as their applications [1–4]. The structure of these compounds loosely resembles that of a tree without its trunk, however they are much more symmetric. The framework of a dendrimer consists of three components: the core, the branches and the surface groups. The number of times that the branching occurs is referred to as the Generation number. In this study, the type of dendrimer used was the poly(amidoamine) or PAMAM dendrimer [5–7], the structure of PAMAM Generation 4 (G4) is shown in Figure 1. PAMAM dendrimers have a hollow core and a densely packed outer layer. PAMAM dendrimers are commercially available. The set used in this work contains an ethylenediamine-based core; the surface groups are amines. As the size of the dendrimer increases, the three-dimensional structure changes. It is known that PAMAM dendrimers of Generations 0–2 have an open structure, sometimes called “starfish-like” [6]. In contrast, dendrimers of Generations 4 and higher are known to be more spherical and closely packed in structure. Generation 3 dendrimers have a shape that is not quite spherical, but not as flat as the lower Generations. The increasing size of the branches allows for more isolation of the dendrimer interior as the Generation increases. For example, a hydrophobic core may be protected from aqueous solution by branches that contain hydrophilic surface groups. Because of this ability, dendrimers are often referred to as molecular micelles [8–12].

The symmetric branching of dendrimers creates distinct cavities between the branches. These cavities may be occupied either by solvent or end groups of the dendrimer that have back folded, or in some cases these cavities will accommodate guest molecules [13]. The number and size of the cavities in a dendrimer molecule depend on the Generation, the number of branches at the core of the dendrimer, and the nature of the repeated units. The presence of these cavities can allow “encapsulation” [14] of smaller molecules within the dendrimer framework. Thus, dendrimers have huge potential as supramolecular hosts. Much research is currently being done into exploiting the host ability of dendrimers for use in biological systems. In particular, dendrimers show great potential as drug-delivery vehicles [15–17].

In an aqueous environment, PAMAM dendrimers are able to encapsulate small, hydrophobic fluorescent probe molecules. The inclusion of a fluorescent probe has a significant effect on its fluorescence properties such as intensity, lifetime, and the position of the maximum fluorescence wavelength. Steady state fluorescence has been widely used to study the microenvironment of dendrimeric cavities and the interactions between the host dendrimer and the guest molecule that has been encapsulated, including the binding constant and stoichiometry of the host-guest complex [18–27].

In addition to the use of fluorescent guests to probe dendrimer binding capacities and cavity properties, fluorescent dendrimers have been prepared with fluorescent probes typically attached as end groups [20,28]. Such dendrimers have tremendous potential applications as fluorescent sensors, to indicate the presence and concentration of specific target guests. For example, Grabchev *et al.* have very recently described a Generation 2 PAMAM dendrimer with 16 1,8-naphthalimide fluorescent peripheral groups, which they showed to be a sensitive and selective fluorescent and colorimetric sensor for Li^+^ ions in solution [29]. PAMAM dendrimers have also been used to make other types of molecular sensors. For example; Zhu *et al.* have published a description of PAMAM dendrimer-enhanced DNA biosensors, in this case based on electrochemical measurements [30]. For all of these applications of PAMAM dendrimers as molecular sensors, a better understanding of their binding capacities, cavity properties and host abilities is required.

Bryszewska *et al.* have extensively studied the binding properties of PAMAM dendrimers Generation 4, 5 and 6 (G4, G5 and G6) using the highly polarity sensitive fluorescent probe 1-anilinonaphthalene-8-sulfonate (1,8-ANS) [21–26]. They used a double fluorometric titration technique, in which the fluorescence is studied both as a function of host concentration at fixed guest concentration and as a function of guest concentration at fixed host concentration [23–26], and found that binding of 1,8-ANS in these dendrimers is very complex. They found that there are two distinctive types of binding centers for G4 and G6 PAMAM dendrimers, which they referred to as low affinity and high affinity, and which correspond to surface and core cavities, respectively [25]. However, in a previous publication they observed only one type of binding site for G4 [23]. Furthermore, they found that the binding constant (K_b_) and number of binding sites (n) both changed dramatically with Generation number, with a value of n = 0.5–0.7 in the case of G5 [26]. The results obtained for G5 dendrimers are of particular interest to us, because they suggest the possibility that 2:1 host-guest complexes are being formed between 1,8-ANS and G5 PAMAM dendrimer. It should be possible to study the binding of 1,8-ANS in G5 using a single fluorescence titration approach (guest fluorescence as a function of host concentration) with fitting to a 2:1 host: guest model, which we have successfully used to study the binding of 1,8-ANS in cucurbit[7]uril [31].

**Figure 2.**
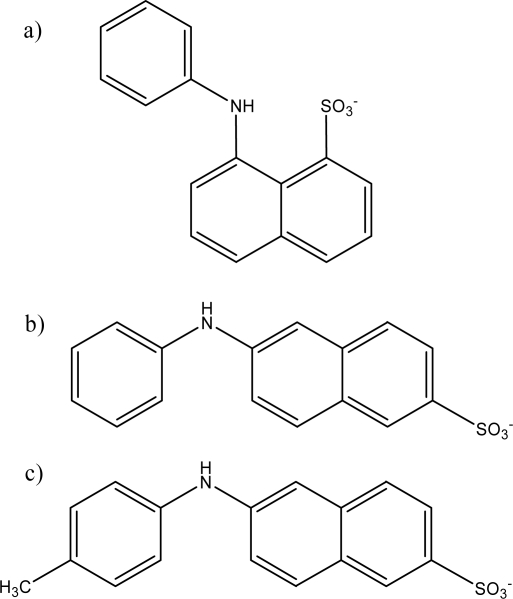
Chemical structure of the three related fluorescent probes used: a) 1,8-ANS; b) 2,6-ANS; c) 2,6-TNS.

In addition, there are other anilinonaphthalene sulfonate fluorescent probes available for studying host properties, with differing shapes, polarity-sensitivity, and electronic properties from those of 1,8-ANS itself, including 2-anilinonaphthalene-6-sulfonate (2,6-ANS) and 6-(*p*-toluidino)-2-napthalene sulfonate (2,6-TNS). The structures of these three probes are shown in Figure 2.

The motivation for this extended ANS study of PAMAM dendrimers is the ability to do comparative binding studies using pairs of closely related probes. Previous work in our research group has involved comparative binding of 1,8-ANS and 2,6-ANS in cucurbit[n]urils [31] and modified cyclodextrins [32]; this allowed for the study of the binding and cavity properties of these important hosts. As can be seen from Figure 2, 1,8-ANS and 2,6-ANS are isomers, differing only in the positions of the anilino and sulfonate groups on the naphthalene fluorophore, but with resultant significant differences in both size and shape, with 2,6-ANS being much longer and more streamlined. For both types of hosts previously studied, the more streamlined 2,6-ANS guests were bound much more tightly than the bulkier 1,8-ANS. We have further expanded this comparative approach in this study by including 2,6-TNS as a probe. The results for 2,6-TNS will be directly compared to the results for 2,6-ANS, as they have very similar shapes, but with 2,6-TNS having a *p*-methyl group attached to the phenyl ring. This will have potential steric and electronic (-CH_3_ is an electron donating group) effects on the guest properties. These comparative binding results will impact the use of these dendrimers as sensors, by determining whether there is a significant effect of the guest shape on its encapsulation within these dendrimers (which would result in a degree of selectivity for the sensor), or whether they can be widely applied as supramolecular hosts for a variety of guest shapes.

This paper thus describes the study of the host properties of PAMAM dendrimers G4, G5 and G6 using this expanded set of anilinonaphthalene sulfonate probes 1,8-ANS (studied previously by Bryszewska *et al.* as described above), 2,6-ANS and 2,6-TNS. These properties are investigated using both a host fluorescent titration approach with fitting to a 2:1 dendrimer:guest model (to investigate whether such a complex is formed for any of these host: guest pairs), and preliminary experiments involving the double fluorescence titration approach used previously by Bryszewska *et al.* If a 2:1 complex is indeed formed for any of these host-guest pairs, then the host fluorescent titration approach would provide a simpler technique for studying the complexation process.

## 2. Results and Discussion

### 2.1. Spectral Shifts and Fluorescence Enhancement of ANS Probes by PAMAM Dendrimers

**Figure 3.**
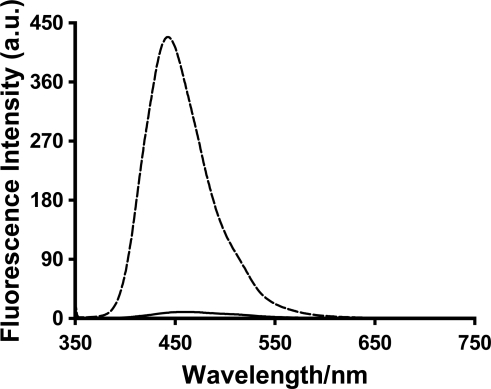
Fluorescence spectra of 4 × 10^−5^ M 2,6-ANS and in the absence (──) and in presence of 1 mM PAMAM dendrimer G5 (----).

A large increase in the fluorescence intensity was observed for all three probes 1,8-ANS, 2,6-ANS and 2,6-TNS upon addition of the various generations of PAMAM dendrimers. The relative fluorescence spectrum of 2,6-ANS in the absence and presence of dendrimer G5 at 1 mM concentration is shown in Figure 3.

The fluorescence enhancement can be quantified as F/F_o_, the integrated fluorescence spectrum in the presence divided by the absence of host. F/F_o_ values at 1 mM dendrimer concentration for the three probes in the Generation 4, 5, and 6 dendrimers are listed in Table 1. These large enhancements indicate that the probes are experiencing significantly reduced polarity environments in the presence of the dendrimer hosts, indicating they have become included within the host interior. The order of enhancement observed of 2,6-TNS > 1,8-ANS ≥ 2,6-ANS (the enhancement for 1,8-ANS is significantly larger than that for 2,6-ANS only in the case of PAMAM dendrimer G6) is a reflection of the differing polarity sensitivity of these three guests, as measured in our group using the Polarity Sensitivity Factor (PSF), which we defined as the ratio of the fluorescence of a probe in ethanol as compared to water [32–34]. We have measured the PSF to be in the same order of 2,6-TNS > 1,8-ANS > 2,6-ANS; thus the differences in enhancement observed is not a reflection of different polarity sites accessed by the three probes. There is also very little difference in the observed values of the enhancement for each probe as a function of Generation number. It can thus be concluded that even with the differences in cavity shape and size for PAMAM dendrimers of different generation number, the local polarity experienced by these guests is similar for each of the three hosts G4, G5 and G6. These hosts have very different diameters of 40, 52 and 67 Å for G4, G5 and G6, respectively [35]. In comparison, 1,8-ANS has a maximum length of *ca.* 11 Å, based on its crystal structure [36], so that it is unlikely that it is fully included within the G4 dendrimer, as was pointed out by Bryszewska *et al.* [23]. The lack of dependence of the observed enhancement on generation number can thus either be a result of the fact that polarity of the cavities is similar for each generation number (since the chemical compositions are the same), or it may indicate a similar degree of encapsulation of the guests into the different generation dendrimer hosts, despite the large difference in host and cavity sizes. In either case, the hydrophobic interaction of the guest and host is seen to contribute to the host-guest inclusion process. In addition, electrostatic interactions between the sulfonate group and protonated terminal amines of the hosts also play a role in the host-guest interactions [23].

There also was a significant blue-shifting of the spectrum of each of the three probes in the presence of all three dendrimers; again this indicates that the probes experience a significantly lower polarity local environment, indicative of inclusion within the host interior. Similarly to the enhancement results, the blue shifts seen for each probe were nearly independent of generation number: 1,8-ANS shifted from 516 nm in the absence of dendrimer to 480, 480 and 478 nm in the presence of 1 mM G4, G5 and G6, respectively; 2,6-ANS shifted from 461 to 444, 442, and 444 nm and 2,6-TNS shifted from 463 to 454, 454, and 453 nm under these same conditions. These results again indicate that similar environments are experienced by the three probes regardless of the generation number.

### 2.2. Comparative Binding of ANS Probes by PAMAM Dendrimers

The increase in the fluorescence intensity and blue shift in the spectra indicates the formation of the host-guest inclusion complexes of each of these three probes with each of the three dendrimers. Multiple single fluorescence titration experiments, the measurement of the fluorescence enhancement (F/F_o_) as a function of host concentration, were performed for each of the 9 host-guest pairs. This data can then be used to extract the binding constant(s), based on a particular complexation model [34]. In the case of simple 1:1 host: guest complexation (which is not expected for dendrimer inclusion, based on the work of Bryszewska *et al.*), the following equation can be fit to extract the binding constant K [37]:

(1) $$\frac{F}{F_{o}}=1+(F_{\text{max}}/F_{o}-1)\frac{K[\mathrm{Host}]}{1+K[\mathrm{Host}]}$$

A double reciprocal plot of 1/(F/F_o_−1) *vs.* [host] can be used to test for simple 1:1 complexation: this plot will be linear if only 1:1 complexation is occurring, but will be non-linear if any other stoichiometry or set of stoichiometries are being formed. As mentioned in the introduction, Bryszewska *et al.* have reported the number of binding sites for 1,8-ANS complexed by PAMAM dendrimers as 0.31 for G4 [23] and 0.5–0.7 for G5 [26]. Thus, more than 1 host is involved in the complexation of a single 1,8-ANS in these two cases, which suggest that a 2:1 host: guest model would be more appropriate for G5 (and perhaps G4) binding of these probes. The following equation has been derived for the stepwise formation of a 1:1 followed by addition of a second host to form a final 2:1 complex [38]:

(2) $$\frac{F}{F_{o}}=\frac{1+\frac{F_{1}}{F_{o}}K_{1}[\mathrm{Host}]+\frac{F_{2}}{F_{o}}K_{1}K_{2}{\left[\mathrm{Host}\right]}^{2}}{1+K_{1}[\mathrm{Host}]+K_{1}K_{2}{\left[\mathrm{Host}\right]}^{2}}$$

**Figure 4.**
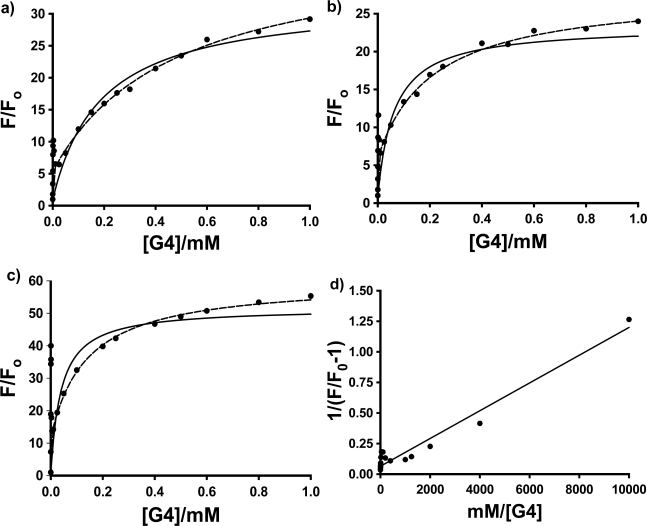
Averaged host fluorescence titration plots for 4 × 10^−5^ M a) 1,8-ANS; b) 2,6-ANS and c) 2,6-TNS as function of PAMAM dendrimer G4 concentration. The double reciprocal plot for 1,8-ANS in G4 is shown in d). The fits to the 1:1 (──) and 2:1 (- - -) models are also shown in parts a to c.

In this equation, K_1_ and K_2_ are the equilibrium constants for the first then second host forming a complex with the guest; F_1_/F_o_ is the enhancement when every guest is bound by 1 host, and F_2_/F_o_ is the enhancement when every guest is bound by two hosts. Figure 4 shows the fluorescence titration curves (based on the average of 3 to 4 individual trials) for the three probes in PAMAM dendrimer G4, with the fits to these two models using non-linear least squares fitting to Equation 1 and 2.

The most interesting and surprising aspect of these titration curves shown in Figure 4 is the anomalous narrow peak at very low [G4], which appears as a spike very close to the y-axis. This intriguing and important phenomenon will be discussed in detail in Section 2.3. It is clear that the entire titration curves, including the spikes at low dendrimer concentration, will not fit to the conventional 1:1 and 2:1 models described above. In order to obtain at least a qualitative indication of the comparative strength of the binding of each probe with G4, fits were therefore performed on the fluorescence titration data omitting the points in the low dendrimer concentration that define the peak. The results are indicated in Figure 4 as solid lines (1:1 fit) and dashed lines (2:1 fit). Also shown is an example of the full double-reciprocal plot (for 1,8-ANS). It is clear from the 1:1 fit lines and the non-linear double reciprocal plot that the 1:1 model is inadequate for these complexes, even with the anomalous peak data omitted, as expected. Figure 4 shows that the 2:1 model does provide a very good visual fit for all three probes in G4, with excellent agreement between the fit line and data points. This is equivalent to 0.5 binding sites per G4 host, compared to the results obtained by Bryszewska *et al.* of 0.31 binding sites for G4 [23]. However, the values of the 4 fit parameters for Equation 2: F_1_/F_o_, K_1_, F_2_/F_o_ and K_2_ were found to vary widely for the individual trials, and no consistent values could be obtained in order to do a quantitative comparison. This could be a result of a number of factors, including the additional complexity indicated by the peaks in the titration curves being manifested throughout the concentration range of the titration data, the lack of points at the important low dendrimer concentration (which were removed because of the spike), and the extremely strong binding, which makes extraction of the binding constants more difficult. Alternatively, this could indicate that simple 2:1 complex formation is not in fact occurring, and that the observed spike is indicative of a much more complex system. Thus, a quantitative comparative binding study could not be performed, but only a qualitative comparison can be made, based on the relative curvature of the titration plots themselves. This can be done by considering the binding constants obtained using the 1:1 model; although clearly being an insufficient fit model, it does provide a direct measure of the curvature of the plots. Relatively consistent results were obtained between trials, and the resulting average values of K can be used to give an indication of the relative binding of the three ANS guests. The results are shown in Table 2; qualitatively at least, 2,6-ANS and 2,6-TNS bind more strongly than 1,8-ANS, indicating that the more streamlined 2,6-substituted guests are a better match for the size and shape of the G4 cavities.

**Figure 5.**
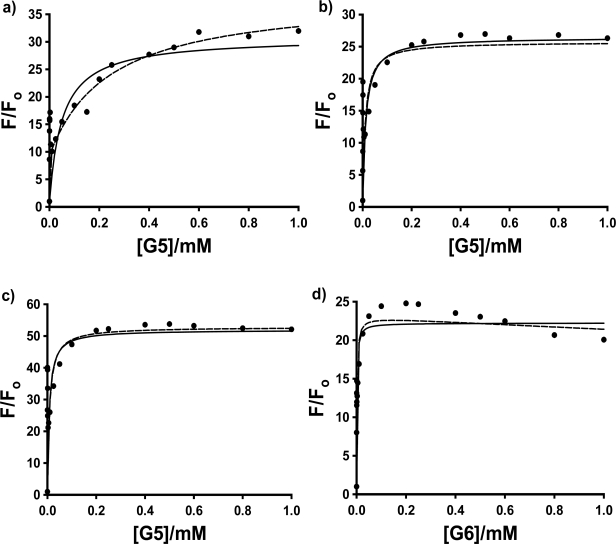
Averaged host fluorescence titration plots for 4 × 10^−5^ M a) 1,8-ANS, b) 2,6-ANS and c) 2,6-TNS as a function of G5 and d) 2,6-ANS as a function of G6 PAMAM dendrimer concentration, with the fits to the 1:1 (──) and 2:1 (- - -) models also shown.

In the case of G5, shown in Figure 5 a to c, the peak in the fluorescence titration data was again present for all three probes, while in the case of G6, the spike was observed consistently in the case of 2,6-ANS and 2,6-TNS, and in 3 of 5 trials in the case of 1,8-ANS. (We have no explanation at this time as to why the spike was not observed in the first two trials performed for 1,8-ANS in G6, especially since the same bottle of G6 dendrimer in methanol was used for the trials with 2,6-ANS and 2,6-TNS, all of which exhibited the anomalous spike.) In these cases, the 2:1 model provided a good visual fit to the titration data only in the case of 1,8-ANS in G5. While this is in agreement with the results of Bryszewska *et al.* of approximately 0.5 binding sites per G5 host [26], once again the fit values of K_1_ and K_2_ were not reproducible from trial to trial, and no quantitative results could be obtained. In the case of G5, the average results for the fits to the 1:1 model are given in Table 1. Once again the 2,6 substituted guests bind much more strongly to G5 than does 1,8-ANS. In addition, 2,6-TNS was found to bind much more strongly than 2,6-ANS, indicating an effect of the methyl substitution on the electronic properties of the naphthalene moiety, and its interaction with the G5 host. In the case of G6, as shown by the representative sample of 2,6-ANS in Figure 5d), not even qualitative comparisons could be made, due to the very steep rise in the titration curves in all three cases.

As a result of the lack of reproducible fit parameters of the fluorescence titration data for the three probes in the three PAMAM dendrimers with either a simple 1:1 or 2:1 host: guest inclusion model, the more complicated double fluorometric titration method used by Bryszeska *et al.* for studying the binding of 1,8-ANS in PAMAM dendrimers [23–27] was undertaken for the three probes. A preliminary set of single trials was performed in G4 as a representative dendrimer host, to do the desired comparative binding study. This approach, as mentioned earlier, involves the measurement of a pair of fluorescence titration curves, one with fixed guest concentration and varying host concentration (equivalent to those shown in Figures 4 and 5), and the other with fixed host concentration and varying guest concentration. In the present study, two separate sets of experiments were done, one with dendrimer concentrations fixed at a higher concentration above the anomalous spike (0.20 mM), and the other with the dendrimer concentration fixed at a low concentration at which the anomalous spike was observed (0.0025 mM). The results from the low dendrimer concentration study will be discussed in the next section.

In this technique, described in detail in the literature [23–27 and references therein, 39], the host titration allows for the determination of the fluorescence enhancement for the bound ANS guest. This value can then be used with the guest titration data to calculate the concentration of free and bound ANS based on the enhancement observed at each guest concentration. The following relationship between the bound and free guest ANS concentrations can then be used to determine the values of the binding constant K_b_ and the number of binding sites n [23–27,39]:

(3) $$\frac{\left[\mathrm{Host}\right]}{{\left[\mathrm{Guest}\right]}_{\mathrm{bound}}}=\frac{1}{K_{b}n{\left[\mathrm{Guest}\right]}_{\mathrm{free}}}+\frac{1}{n}$$

Thus, a double-reciprocal plot of [host]/[guest]_bound_ *vs.* 1/[guest]_free_ should yield a straight line, with values of n and K_b_ obtainable from the y-intercept and the slope.

**Figure 6.**
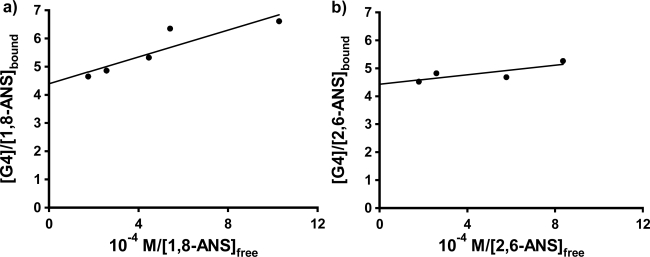

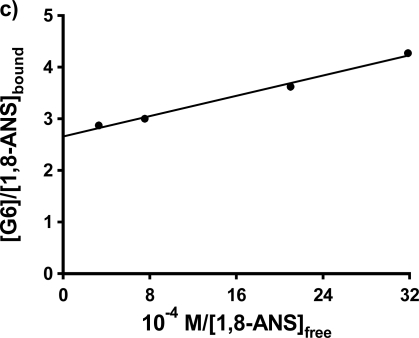
The double-reciprocal plots from the guest titration data for 4 × 10^−5^ M (a) 1,8-ANS and (b) 2,6-ANS in the presence of 0.20 mM G4 PAMAM dendrimer, and (c) 2,6-TNS in the presence of 0.20 mM G6 dendrimer; the solid lines shows the linear fits.

Figure 6a and b shows these plots for 1,8-ANS and 2,6-ANS in the presence 0.20 mM G4 dendrimer, respectively (well above the host concentration at which the anomalous spike was observed in each case). The results for the fits to Equation 3 are shown in Table 3. For 1,8-ANS with G4, Bryszewska *et al.* reported K_b_ values of 5.6 × 10^4^ M^−1^ [23], 2.6 × 10^5^ M^−1^ (low affinity binding centers) and 3.7 × 10^6^ M^−1^ (high affinity binding centers) [25] and n values of 0.31 [23], 0.60 (low affinity binding centers) and 0.34 (high affinity binding centers) [25] in buffer solution. Our value of K_b_ of 1.8 ± 0.5 × 10^5^ in nanopure water is in best agreement with that reported for the low affinity (surface) binding centers, however our value of n is slightly lower than that reported. For 2,6-ANS with G4, a higher constant was obtained than that for 1,8-ANS, in agreement with the qualitative results obtained from the 1:1 fits above that the more streamlined 2,6-ANS binds more strongly to G4 PAMAM dendrimers than does the comparatively bulky 1,8-ANS. In the case of 2,6-TNS in G4, the resulting double reciprocal plot was too highly scattered to allow for the determination of K_b_ and n.

Single trial double fluorometric titrations were also carried out for 1,8-ANS in G5 and G6 at 0.20 mM host concentrations. In the case of G5, the resulting double reciprocal plot was found to be curved, so that the values of K_b_ and n could not be determined. In the case of 1,8-ANS in G6, the double reciprocal plot was linear, as shown in Figure 6c); the fit results are listed in Table 3. The value of K_b_ obtained falls between the two values reported for 1,8-ANS in G6 in buffer solution by Bryszewska *et al.* [25] for the low and high affinity binding centers. However the value of n obtained here is very small, and is expected to be much greater than 1 for this very large dendrimer [25]. Future work will involve more double fluorescence titration experiments to further delineate the current results using 1,8-ANS, in addition to investigating the comparative binding of 2,6-ANS and 2,6-TNS in these hosts.

### 2.3. The Unusual Fluorescence Titration Behaviour at Low Dendrimer Concentration

**Figure 7.**
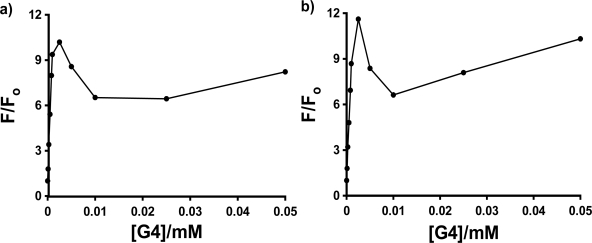
Expansion of the low host-concentration region of the averaged host fluorescence titration plots for 4 × 10^−5^ M a) 1,8-ANS and b) 2,6-ANS as a function of G4 PAMAM dendrimer concentration {from Figure 4 a and b)}, illustrating the anomalous spike in fluorescence enhancement.

As was seen in Figures 4 and 5, there is a sharp spike in the measured fluorescence enhancement in the host titration curves, followed by a more expected smooth increase in fluorescence with increasing concentration of the dendrimer host. This anomalous phenomenon was found to occur consistently for all three anilinonaphthalene sulfonate probes in all three PAMAM Generation 4–6 hosts, with the exception of 1,8-ANS in G6, in which case the spike was observed in three of five host fluorescence titration trials. The spike is more clearly shown in Figure 7, which shows an expansion of the host titration curve at very low PAMAM dendrimer concentration for the case of 1,8-ANS and 2,6-ANS in G4 host. In all cases, there was no difference in the wavelength of maximum emission in the spectra at low concentration (where the spike was observed) compared to high concentration, which indicates that the guest experiences a similar environment at these two concentrations.

The enhancement of the peak at low host concentrations is quite significant in comparison to the enhancement at the higher host concentrations. This can be quantified as the ratio of the fluorescence enhancement F/F_o_ measured at the height of the low concentration peak to that measured at the highest dendrimer concentration (1 mM in all cases). In the case of G4 host, the fractional height of the peak was found to be 0.35, 0.48 and 0.72 for 1,8-ANS, 2,6-ANS and 2,6-TNS, respectively. A similar pattern of increasing relative size of the peak was also observed for the three probes in G5 (0.54, 0.74 and 0.77). In the case of G6, while the peak was not consistently observed for 1,8-ANS, a large peak was observed for 2,6-ANS (0.65), and the peak for 2,6-TNS was in fact observed to be larger than the enhancement at 1 mM, with a fractional size of 1.3. Thus, the formation of the anomalous peak is facilitated by the more streamlined shape of the 2,6-substituted guests.

**Figure 8.**
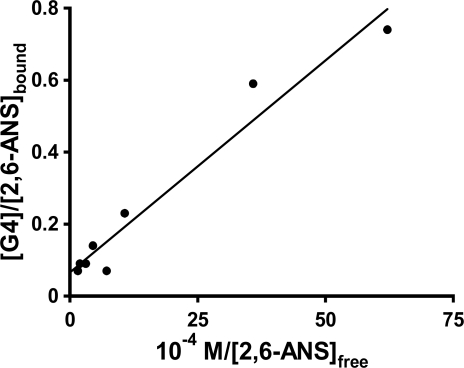
The double-reciprocal plot from the guest titration data for 4 × 10^−5^ M 2,6-ANS in the presence of 0.0025 mM G4 PAMAM dendrimer; the solid line shows the linear fit.

In order to further investigate the nature of the interactions between the ANS guests and the PAMAM dendrimers which results in these spikes in the host fluorescence titration plots, single trial double fluorometric titration studies were done for the three probes in G4, again as a representative dendrimer host for comparative binding studies, as well as for 1,8-ANS in G5 and G6. The guest fluorescence titration plot was carried out at the concentration of the maximum observed anomalous peak in the host fluorescence titration plot ([G4] = 0.0025 M in all cases). In the case of 1,8-ANS in both G4 and G5, the resulting double reciprocal plots yielded negative y-intercepts (but close to 0), which has no physical meaning (n, the number of binding sites, would be negative). In the case of both 2,6-TNS in G4 and 1,8-ANS in G6, the double reciprocal plot was highly scattered, preventing meaningful determination of n and K_b_. However, in the case of 2,6-ANS, the double reciprocal plot was reasonably linear, as shown in Figure 8, and gave a very small but positive y-intercept, allowing for the determination of n and K_b_.; these are listed in Table 3. The values of K_b_ = 5.9 ± 2.5 × 10^4^ M^−1^ and n = 15 ± 6 obtained at the spike region are very different from those described above for 2,6-ANS using guest fluorescent titration at a fixed [G4] = 0.20 M (well above the region of the spike) of K_b_ = 5.3 ± 2.7 × 10^5^ M^−1^ and n = 0.23 ± 0.01. These results indicate that at the [G4] concentration at which the spike occurs, the binding of 2,6-ANS is weaker, but many more guests are bound per G4 host, as compared to higher host concentrations. Thus, there is a significant change in the nature of the binding between the 2,6-guest and the G4 host in these two concentration regimes. However, it is unclear at this point how such a change in the nature of the binding of the host with the guest with changing host concentration would result in the observed spike in the fluorescence enhancement, particularly in light of the similar shape and position of the 2,6-ANS fluorescence spectrum at the various concentrations of host. Future work will be needed to explain the origin of the spike.

This anomalous spike in the host fluorescence titration curve is, to the best of our knowledge, unprecedented in the literature. The only similar report of a fluorescence titration in which an initial steep increase in fluorescence was followed by a sharp decline as a function of host concentration was reported for pyrene as a guest in G2 starburst polymer hosts [6]. However, that fluorescence titration involved pyrene excimer fluorescence, and was explained as a result of the dependence of the formation of pyrene excimers in the host cavity (1:2 or 2:2 host: guest complexes) on host concentration. Excimer formation decreased as the host concentration increased, as inclusion of pyrene guest pairs gave way to inclusion of pyrene monomers. This scenario is not occurring in our ANS-PAMAM dendrimer systems, as these guests do not exhibit excimer fluorescence; the changes in fluorescence intensity are solely a result of the changes in binding and local environment on individual ANS guests.

## 3. Experimental Section

PAMAM dendrimers, ethylenediamine core, Generations 4, 5 and 6 were obtained as solutions in methanol from the Aldrich Chemical Company and stored at 4 °C. Generations 4, 5 and 6 contain 64, 128 and 256 surface amine groups, respectively. The fluorescent probes 1,8-ANS and 2,6-TNS were also purchased from Aldrich. The probe 2,6-ANS was obtained from Molecular Probes, Inc. All chemicals were used as received.

Stock solutions of the fluorescent probes were prepared from ultrapure distilled, deoinized water at a concentration of 4 × 10^−5^ M, giving absorbances between 0.2 and 0.4 at the excitation wavelength of 340 nm. Samples were prepared by removing the methanol from the dendrimer solution using a steady flow of argon. After removing the methanol, the desired amount of probe solution was added to the remaining film of dendrimer. The sample was then sonicated to ensure thorough mixing of dendrimer and probe. All absorbance and fluorescence measurements were performed on solutions in 1 cm^2^ quartz cuvettes. Absorbance measurements were obtained using a Varian Cary 50 Bio UV-vis spectrophotometer. Fluorescence spectra were measured on Perkin-Elmer Fluorescence Spectrophotometer.

The fluorescence enhancement was determined by calculating the ratio (F/F_o_) of the integrated fluorescence intensity of the fluorescent probe in the presence (F) and absence (F_o_) of the dendrimer, corrected for solvent background emission. Fits of the host fluorescence titration curves to 1:1 and 2:1 models were performed using non-linear least squares fitting programs for each model, written in our laboratory. The double fluorescence titration data was calculated and analyzed according to equations found in the literature [23].

## 4. Conclusions

In this work the abilities of PAMAM dendrimers of Generation 4–6 as supramolecular hosts were studied. The fluorescent probes 1,8-ANS, 2,6-ANS and 2,6-TNS all form host-guest inclusion complexes with these dendrimers, with significant enhancement of their fluorescence upon complexation. However, in all cases, the complexation is very complicated, and the host fluorescence titration plots could not be fit using simple 1:1 or 2:1 host: guest models. Also, in all cases a very interesting and unique spike in the host titration plots was observed at low host concentration. Preliminary double fluorometric titration analysis indicated that there is a significant difference in the binding of 2,6-ANS by G5 at low as compared to high host concentration, with many guests per host bound at low host concentration, but less than one guest per host at higher concentration. This dramatic change in the nature of the host-guest complexation as a function of host concentration explains why a simple 2:1 model for the host fluorescence titration data was not sufficient for fitting the titration data.

The use of this set of related fluorescent probes as guests allowed for comparative binding studies, which provided information about the nature of the dendrimer host cavities. Both the complete qualitative comparison of the host fluorescence titration plots (omitting the spikes) for PAMAM dendrimers G4 and G5 and the preliminary double fluorometric titration method in the case of PAMAM dendrimer G4 indicated that the more streamlined 2,6-ANS is more tightly bound than the more bulky 1,8-ANS, suggesting that the cavities in these dendrimers best accommodate narrow, longer guests.

These PAMAM dendrimers were shown to be very complicated supramolecular hosts (as is well known in the literature), with large variations in host properties dependent on guest size and shape, as well as the dendrimer host concentration. They exhibit different host behavior at low and high concentration, which is manifested in unusual host fluorescence titration curves. This complex behavior has implications for the use of PAMAM dendrimers as hosts, for example in fluorescent sensor applications for specific target guests. In such applications, great care would need to be taken to ensure that the concentration of dendrimer used was on the plateau region of the fluorescence titration curve, and not in the region of this anomalous peak, as the sharp nature of the peak could result in large changes in fluorescence intensity from sample to sample, with small possible variations in the dendrimer host concentration. In this way, the anomalous fluorescence titration behavior observed at low dendrimer concentration will not impact the proven utility of these PAMAM dendrimers as supramolecular hosts in sensor applications. Future work will expand upon the preliminary double fluorometric experiments reported herein, and will involve complete sets of experiments with multiple trials for each of the nine ANS-dendrimer host: guest pairs, to provide a more detailed comparative binding study and a deeper understanding of the source of the host fluorescence titration spike.

## Figures and Tables

**Table 1.** The fluorescence enhancements of 4 × 10^−5^ M 1,8-ANS, 2,6-ANS and 2,6-TNS by 1 mM PAMAM dendrimers G4 to G6.

**Generation**	**1,8-ANS**	**2,6-ANS**	**2,6-TNS**
**G4**	31 ± 9	26 ± 3	55 ± 2
**G5**	34 ± 7	26 ± 6	52 ± 8
**G6**	32 ± 6	20 ± 2	59 ± 2

**Table 2.** The average association constant K from the 1:1 model fit results for 4 × 10^−5^ M 1,8-ANS, 2,6-ANS and 2,6-TNS in 1 mM PAMAM dendrimers G4 and G5.

	**K/M^−1^**		

**Generation**	**1,8-ANS**	**2,6-ANS**	**2,6-TNS**
**G4**	5.8 ± 1.8 × 10^3^	1.6 ± 0.3 × 10^4^	2.3 ± 0.4 × 10^4^
**G5**	1.9 ± 0.9 × 10^4^	4.5 ± 2.2 × 10^4^	1.3 ± 0.1 × 10^6^

**Table 3.** Results for the fits of the double fluorometric titration experiments to Equation 3.

**Guest**	**Host Generation (Concentration/mM)**	**n**	**K_b_/M^−1^**
**1,8-ANS**	**G4 (0.20 mM)**	0.23 ± 0.02	1.8 ± 0.5 × 10^5^
**2,6-ANS**	**G4 (0.20 mM)**	0.23 ± 0.01	5.3 ± 2.7 × 10^5^
**1,8-ANS**	**G6 (0.20 mM)**	0.38 ± 0.0	5.4 ± 0.4 × 10^5^
**2,6-ANS**	**G4 (0.0025 mM)**	15 ± 6	5.9 ± 2.5 × 10^4^
